# An Ultra-High Element Density pMUT Array with Low Crosstalk for 3-D Medical Imaging

**DOI:** 10.3390/s130809624

**Published:** 2013-07-26

**Authors:** Yi Yang, He Tian, Yu-Feng Wang, Yi Shu, Chang-Jian Zhou, Hui Sun, Cang-Hai Zhang, Hao Chen, Tian-Ling Ren

**Affiliations:** 1 Institute of Microelectronics, Tsinghua University, Beijing 100084, China; E-Mails: yiyang@tsinghua.edu.cn (Y.Y.); tianh10@mails.tsinghua.edu.cn (H.T.); wang-yf05@mails.tsinghua.edu.cn (Y.-F.W.); shuyithuphy@gmail.com (Y.S.); zhoucj86@gmail.com (C.-J.Z.); sophiesun1987@163.com (H.S.); canghaizh@hotmail.com (C.-H.Z.); haochen@Princeton.edu (H.C.); 2 Tsinghua National Laboratory for Information Science and Technology (TNList), Tsinghua University, Beijing 100084, China

**Keywords:** piezoelectric, ultrasonic transducer, Si-SOI bonding, IC integration compatibility, 3-D medical imaging

## Abstract

A ∼1 MHz piezoelectric micromachined ultrasonic transducer (pMUT) array with ultra-high element density and low crosstalk is proposed for the first time. This novel pMUT array is based on a nano-layer spin-coating lead zirconium titanium film technique and can be fabricated with high element density using a relatively simple process. Accordingly, key fabrication processes such as thick piezoelectric film deposition, low-stress Si-SOI bonding and bulk silicon removal have been successfully developed. The novel fine-pitch 6 × 6 pMUT arrays can all work at the desired frequency (∼1 MHz) with good uniformity, high performance and potential IC integration compatibility. The minimum interspace is ∼20 μm, the smallest that has ever been achieved to the best of our knowledge. These arrays can be potentially used to steer ultrasound beams and implement high quality 3-D medical imaging applications.

## 1. Introduction

Ultrasonography is an indispensable method for medical imaging. Its importance shows in several ways. Firstly, it is inexpensive compared with other imaging techniques (CT, PET or MRI); secondly, it is harmless to the patient in medical practice; thirdly, with ultrasonography it is easy to get real-time imagery [1–5]. Advances in electronics over the years have brought extraordinary improvements to all the parts of ultrasound imagers but little to the transducer [6–9]. Regarding the existing 2-D transducer arrays, some of their significant challenges include complex fabrication, high manufacture cost and bulky volume [10–12]. Commercial 2-D transducer probes are bulky structures and typically limited to arrays with an element pitch of 100 μm. The large pitch and scale of the transducer array limits the operation frequency, which will heavily influence the axial and lateral resolution of the ultrasonic imaging. With the development of MEMS, piezoelectric micromachined ultrasonic transducers (pMUT) [13–15] have become a very promising alternative to replace traditional bulk ceramic transducers. Some pMUT prototypes with miniaturized size, good performance and excellent consistency have been reported [16–18]. However, for the existing pMUT structure, it is still difficult to fabricate compact arrays. The smallest interspace of extant pMUT arrays is about 30 μm achieving by DRIE from the back through the whole wafer [19]. The element density has thus become the bottleneck to the development of pMUT arrays. Increasing the element density is crucial to realize 2-D pMUT arrays for practical 3-D medical imaging. Moreover, the crosstalk between different elements of the array should be controlled while reducing the interspace [20].

In our previous work, we reported on the manufacture of 6 × 6 pMUT arrays with element pitches down to 250 μm [21,22] and 150 μm [23] using a conventional silicon wet-etching method. Recently, we also reported some initial results on the reverse-bonding structure [15]. However, there is no report on device performance and simulation.

In this paper, a ∼1 MHz pMUT array based on an Si-SOI wafer bonding technique with ultra-high element density and low crosstalk is proposed for the first time. The minimum interspace is ∼20 μm. The layer-by-layer spin-coating nano-thickness lead zirconium titanium (PZT) film technique is feasible to obtain high frequency.

## 2. Experimental Section

**Figure 1.**
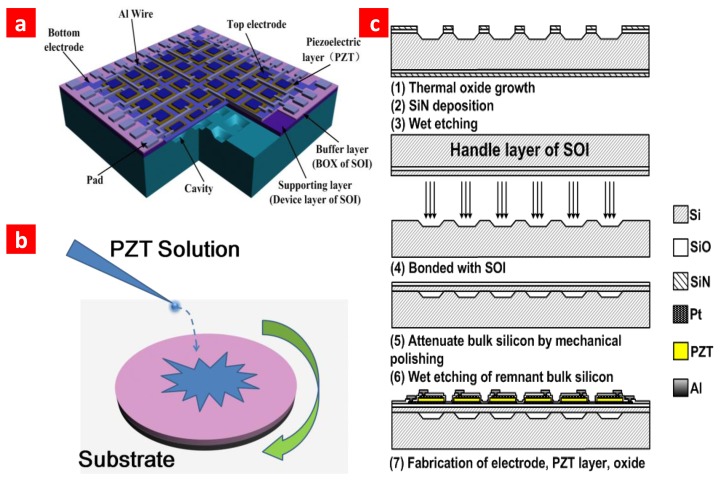
Schematic diagram of the pMUT device structure and fabrication process. (**a**) Schematic diagram of the novel pMUT array based on the SOI-bonding technique. A thin layer of SiO2 is deposited on the top by PECVD as a passivation layer (not shown in the diagram). Pt is used as electrode. To increase the sensitivity, it only covers part of the piezoelectric layer. (**b**) The schematic diagram of spin-coating a PZT nano-layer. The thickness of each nano-layer is estimated at 100 nm. The thick PZT film could be fabricated by layer-by-layer spin-coating PZT solution. (**c**) The whole fabrication process for the “reverse-bonding” structure.

Figure 1a shows a pictorial diagram of our novel pMUT array. The piezoelectric layer is sandwiched between electrodes. A four-edge clamped membrane structure is designed to develop a high-frequency device. A schematic depiction of spin-coating PZT film is shown in Figure 1b. Thickness parameters of the structure are listed in Table 1. Each layer is estimated to be 100 nm. As previously reported, the 2 μm PZT film can be fabricated by layer-by-layer spin-coating PZT solution [15]. The fabrication process of the pMUT array is shown schematically in Figure 1c. First, high-density cavities were etched by potassium hydroxide (KOH) etching solution. Then the wafer was bonded to the device layer of SOI. To simplify fabrication and reduce residual stress, the Si wafer and SOI were bonded in atmosphere. The maximum temperature the wafer exposed in our process is 900 °C. We could use PT/PZT/PT structure to reduce the temperature to 600–700 °C [24]. This would make this technology compatible for IC integration.

**Figure 2.**
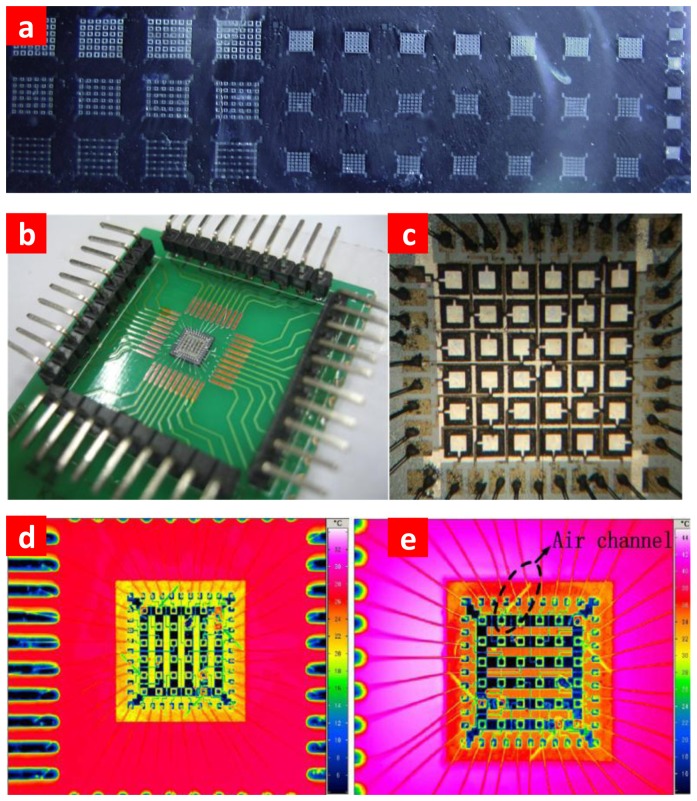
Photograph of pMUT. (**a**) The wafer after SOI bonding. (**b**) Sample used for testing. (**c**) Micrograph of the 6 × 6 pMUT array with element size 200 × 200 μm^2^, the total area is less than 2 × 2 mm^2^. (**d**) Infrared photo of the 6 × 6 pMUT array under room temperature. (**e**) Infrared photo of the 6 × 6 pMUT array heating with a small heater. Air channel stretched to the edge can be seen.

The wafer after SOI bonding is shown in Figure 2a. Photograph of the sample and full view of the high-density array are shown in Figure 2b,c. In order to avoid blowing up during annealing, an air channel structure was designed. To confirm that the air channel is complete and unblocked after fabrication, we used a thermal infrared imager (Image IR) to analyze the underneath structure. The thermal infrared image was captured by a Fluke Ti45. As the thermal conductivity of the air and silicon are different, we can see the air channel stretched to the edge in Figure 2d,e. From the infrared photos shown in Figure 2d,e, the air channel extends to the edge without blocking.

**Figure 3.**
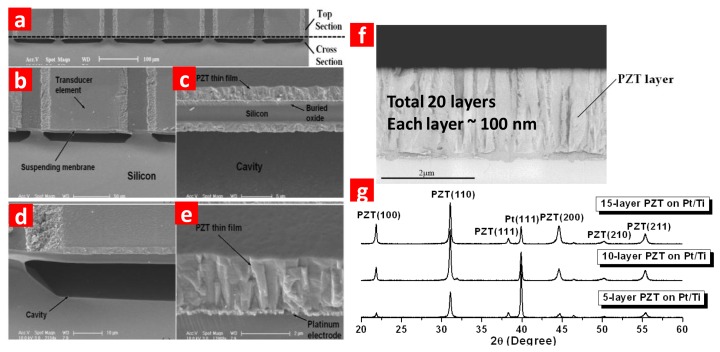
SEM and XRD of the fabricated pMUT array. (**a**) Oblique view of the structure. (**b**) Oblique view of a single element. (**c**) The suspended membrane consisted of multi-layers. (**d**) Oblique view of the cavity structure. (**e**) Detail of the piezoelectric layer. (**f**) SEM photo for thick PZT film. Each nano-layer of PZT is estimated 100 nm. The thick PZT film was fabricated by layer-by-layer spin-coating PZT solution. The prepared PZT film reaches the designed thickness of 2 μm. (**g**) X-ray diffraction result for various numbers of layers. The PZT film shows a well crystallized structure.

SEM photos in Figure 3a–e clearly show the element surface, the multi-layer structure and the ∼20 μm interspace. The fabricated transducers need to be poled before testing. Lead zirconium titanium (PZT) is used as piezoelectric layer. As transduction layer, thicker PZT brings enhanced driving capability to the device and improved sensitivity. SEM photo and XRD result of the PZT film prepared by an optimized sol-gel method are shown in Figure 3f,g.

**Figure 4.**
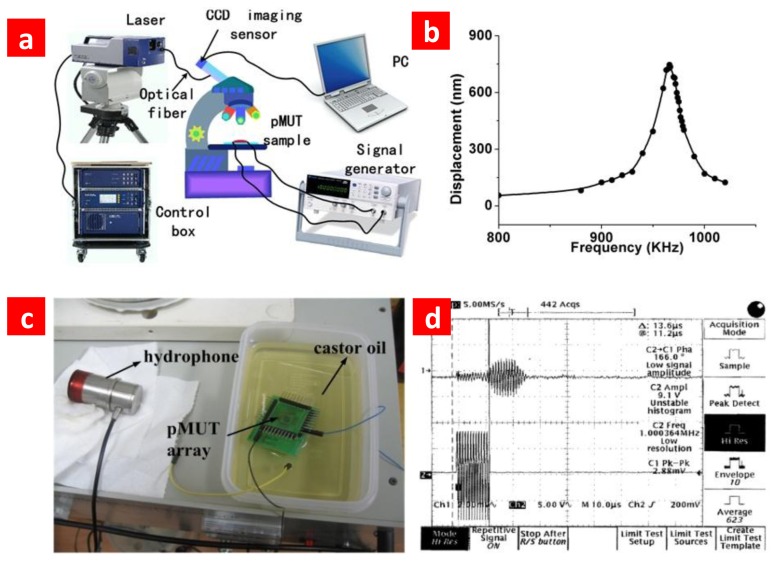
Experimental results of pMUT. (**a**) Vibration test platform. (**b**) Vibration under different signal stimulus (v_pp_ = 4 V). The element length is 200 μm. (**c**) Experiment set for the acoustic test under castor oil. The hydrophone is used to accept the ultrasound transmitted by the pMUT array. (**d**) Testing result. Channel 1 is the received signal and channel 2 is the stimulus signal.

We used a laser vibrometer (Polytec PSV-400) to directly observe the acoustic characteristic of the fabricated arrays, as shown in Figure 4a. Vibration under different signal stimulus is shown in Figure 4b. The resonance frequency acquired by vibration test is well consistent with the result of the impedance analysis. In order to reduce the crosstalk we took some special precautions during the designing of the pMUT array. The PZT was etched to separate each independent element. As a result, according to the vibration testing results the crosstalk between adjacent elements can be neglected. The cross talk is no more than the environment noise, and the SNR of the device while operated under 4 V voltage is about 43 dB. The bandwidth of PMUT with element size 200 × 200 μm^2^ is 50 kHz, which could be estimated through vibration results in Figure 4b. We also tested the functionality of the fabricated pMUT array in a liquid environment, and the experiment set and test result is shown in Figure 4c,d. The resulta show that it can work well under castor oil.

The cross-talk between different elements in the array is a crucial problem. In order to demonstrate the crosstalk effect experimentally, we apply stimulation signal on one element and test the output signal of the adjacent element by vibration testing (Figure 4). We didn't detect obvious output signal from the adjacent element. The output of the adjacent element has no different with the noise signal when we didn't apply any input signal. That means the cross-talk can be neglected.

## 3. Results and Discussion

**Figure 5.**
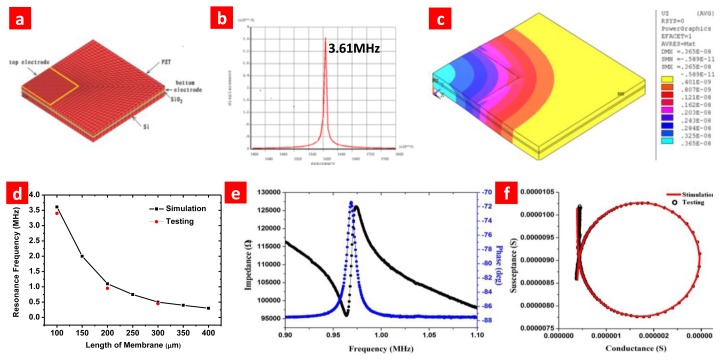
Numerical simulation with ANSYS. (**a**) ANSYS model for the pMUT, a quarter is enough due to the symmetry of the structure. (**b**) Harmonic analyze of the model which shows the resonance frequency is about 3.611 MHz (the element size is 100 μm × 100 μm, other parameters are shown in Table 1). (**c**) The vibration amplitude of the PZT film. (**d**) Comparison of simulation results with experimental results. The experimental results fit well with the simulation. (**e**) Impedance spectrum shows the pMUT with element length 200 μm has electromechanical coupling coefficient of 2.15%. (**f**) Admittance chart shows the difference between the test results and fitting results. Q value of the same transducer obtained from the fitted equivalent circuit is 95.9.

For a 2-μm thick PZT film, its resonance frequency at d_33_ mode should be about 500 MHz to 1 GHz. Our PZT is resonant at d_31_ mode, which is related to a few MHz. In order to demonstrate this, we make numerical simulation with ANSYS. As shown in Figure 5a, an ANSYS model was established for the pMUT. As shown in Figure 5b, harmonic analysis of the model in d_31_ mode shows the resonance frequency is about 3.611 MHz (the element size is 100 μm × 100 μm, other parameters are shown in Table 1).

The thickness of each layer is modeled by the finite element method (FEM) to make a trade-off between transmitting ultrasonic pressure and receiving sensitivity. Element size is used to achieve different resonance frequencies. The FEM model and harmonic analysis of a pMUT element with element size 100 μm × 100 μm are shown in Figure 5a,b. The vibration amplitude of the PZT film is shown in Figure 5c. Capacitances of elements with different size are listed in Table 2. Their capacitances are considerably high compared with that of bulk ceramic transducer which are normally less than 10 pF. Impedance analysis is used to measure the resonance frequency (f_r_) of transducer. Impedance spectrum is shown in Figure 5d,e. The fr is around 1 to 3.6 MHz and can meet the requirement of medical imaging application well [25]. The experimental results fit well with the simulation as Figure 5f shows. Coupling coefficient k_eff_ and quality factor Q can be extracted by equivalent circuit. The fabricated pMUT have relatively high keff and Q. The PZT Stiffness coefficient and piezoelectric matrix parameter are listed in Table 3. The main parameters used in the simulation are listed in Table 4.

**Figure 6.**
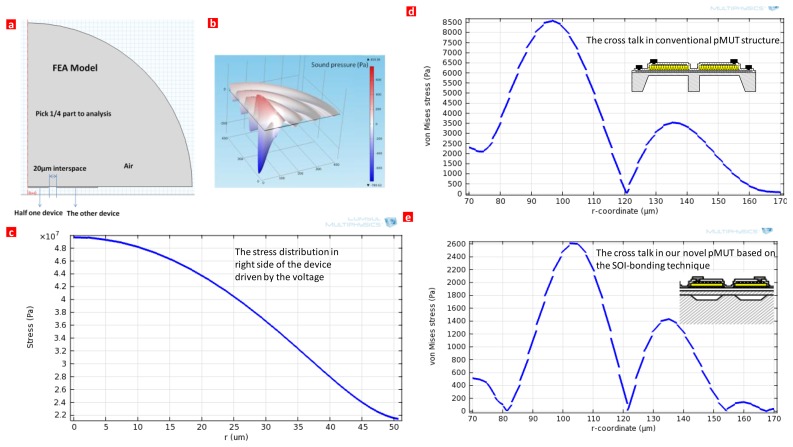
Numerical simulation the cross talk effect in pMUT. (**a**) FEA model for the cross talk in pMUTs, left one is driven by the voltage to see the stress distribution in the right one device. A quarter is enough due to the symmetry of the structure. (**b**) Sound pressure distribution in the air. (**c**) The stress distribution in the left side of the half one device with maximum stress up to 5 × 10^7^ Pa. (**d**) The cross talk of the conventional pMUT structure with maximum stress to 8.5 × 10^3^ Pa. Inset showing the conventional pMUT structure. (**e**) The cross talk of our novel pMUT structure with maximum stress just to 2.6 × 10^3^ Pa. Inset showing our novel pMUT structure.

The cross-talk effect is simulated theoretically. Figure 6 shows a numerical simulation of the cross talk effect in pMUT. Figure 6a shows a FEA model for the cross talk in the pMUTs. The left one is driven by the voltage to see the stress distribution in the other device. If the stress in the left one device is quite low compared with the right device, we can conclude that the cross talk is minimized. A quarter is enough due to the symmetry of the structure. Figure 6b shows the sound pressure distribution in the air. Figure 6c shows the stress distribution in the left half one device with maximum stress up to 5 × 10^7^ Pa. Figure 6d shows the cross talk of the conventional pMUT structure with maximum stress to 8.5 × 10^3^ Pa. As shown in Figure 6e, the cross talk of our novel pMUT structure with maximum stress was just 2.6 × 10^3^ Pa. Compared to the conventional device, the cross talk effect is reduced more than 3-fold by this design.

## 4. Conclusions

A novel pMUT array with ultra-high element density, low crosstalk and excellent performance was designed and demonstrated for the first time. The nano-layer PZT film is a feasible technique to obtain high frequency. Each layer is estimated at 100 nm. The 2 μm PZT film was fabricated by a nano-layer spin-coating PZT solution. The interspace of pMUT elements is the smallest that has ever been achieved. The developed arrays have ∼1 MHz resonance frequency, a modest quality factor. They can be used to form and steer ultrasound beams with strong directionality and high radiation acoustic power. High-resolution 3-D digital ultrasonic medical imaging system, especially in some cutting-edge areas such as intravascular imaging for clinical evaluation could be implemented using these novel pMUT arrays.

## Figures and Tables

**Table 1.** Thicknesses parameter of the pMUT structure.

**Functions**	**Materials**	**Thickness (μm)**	**Fabrication method**
Wire and pad	Al	1.1	DC sputtering
Passivation layer	SiO_2_	0.3	PECVD
Top electrode	Pt	0.2	RF sputtering
Piezoelectric layer	PZT	2.0	Sol-gel
Bottom electrode	Pt	0.2	RF sputtering
Adhesive layer	Ti	0.03	DC sputtering
Stress buffer	SiO_2_	1	BOX layer of SOI
Supporting layer	Si	3	Device layer of SOI
Bulk silicon	Si	520	4-inch Si wafer

**Table 2.** Measured capacitance and resonance frequency.

**Element length (μm)**	**Capacitance (pF)**	**f_r_ (kHz)**	**Q**	**k_eff_**
100	42	3387	60.97	3.07%
200	152	970	95.94	2.15%
300	385	480	63.61	2.78%

**Table 3.** PZT Stiffness coefficient and piezoelectric matrix parameter

**PZT Thin Film Stiffness Coefficient (GPa)**	c_11_	c_12_	c_13_	c_33_	c_44_
	135	67.4	68.1	113	22.2

**Bulk PZT 4 Stiffness Coefficient (GPa)**	c_11_	c_12_	c_13_	c_33_	c_44_
	132	71	73	115	30

**PZT Thin Film Strain piezoelectric Coefficient (C/m^2^)**	e_31_	e_33_	e_15_		
	−1.86	9	9.8		

**Bulk PZT 4 Strain piezoelectric Coefficient (C/m^2^)**	e_31_	e_33_	e_15_		
	−4.1	14.1	10.5		

**Table 4.** The main parameters used in the simulation.

**Material**	**Modulus of Elasticity (GPa)**	**Poisson Ratio**	**Density(Kg/m^3^)**	**Relative Dielectric Constant**
PZT	/	/	7400	540
Si	120	0.42	2331	12.1
SiO_2_	78	0.17	2250	4.6
Pt	95	0.33	21500	/
TiO_2_	230	0.27	4000	85
